# 2-Benzyl-6-benz­yloxypyridazin-3(2*H*)-one

**DOI:** 10.1107/S1600536811011597

**Published:** 2011-04-07

**Authors:** Zhi-Yu Ju, Wan-Xiang Jiang, Feng-Ling Yang

**Affiliations:** a College of Chemistry and Chemical Engineering, Xuchang University, Xuchang, Henan Province, 461000, People’s Republic of China; b Department of Electromechanical Engineering, Xuchang Institute of Technology, Xuchang, Henan Province, 461000, People’s Republic of China

## Abstract

In the title compound, C_18_H_16_N_2_O_2_, the central pyridazine ring forms dihedral angles of 77.08 (5)° and 84.62 (5)° with the two benzene rings. The dihedral angle between the two benzene rings is 68.18 (4)°. A very weak intra­molecular C—H⋯N hydrogen bond and an intra­molecular C—H⋯π inter­action occur. The crystal structure is stabilized by weak inter­molecular C—H⋯O hydrogen bonds and weak C—H⋯π and π–π stacking inter­actions [centroid–centroid distance = 3.6867 (10) Å].

## Related literature

For applications of pyridazinone analogues as highly selective anti-HIV agents, see: Loksha *et al.* (2007 ▶). For applications as pesticide agents, see: Li *et al.* (2005 ▶); Selby *et al.* (2002 ▶). For applications as herbicides, see: Xu *et al.* (2006 ▶). For related structures, see: Liu *et al.* (2005 ▶).
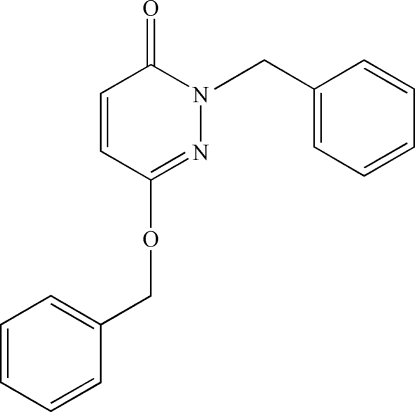


## Experimental

### 

#### Crystal data

C_18_H_16_N_2_O_2_
*M* *_r_* = 292.33
Monoclinic, 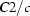

*a* = 32.741 (4) Å
*b* = 10.9198 (14) Å
*c* = 8.1228 (10) Å
β = 95.92 (2)°
*V* = 2888.6 (6) Å^3^
*Z* = 8
Mo *K*α radiation
μ = 0.09 mm^−1^
*T* = 113 K
0.20 × 0.18 × 0.12 mm

#### Data collection

Rigaku Saturn CCD area-detector diffractometer
Absorption correction: multi-scan (*CrystalClear*; Rigaku/MSC, 2009 ▶) *T*_min_ = 0.982, *T*_max_ = 0.989
18031 measured reflections
3448 independent reflections
2142 reflections with *I* > 2σ(*I*)
*R*_int_ = 0.063

#### Refinement

*R*[*F* ^2^ > 2σ(*F* ^2^)] = 0.047
*wR*(*F* ^2^) = 0.116
*S* = 0.95
3448 reflections
199 parameters
H-atom parameters constrained
Δρ_max_ = 0.23 e Å^−3^
Δρ_min_ = −0.23 e Å^−3^

### 

Data collection: *CrystalClear* (Rigaku/MSC, 2009 ▶); cell refinement: *CrystalClear*; data reduction: *CrystalClear*; program(s) used to solve structure: *SHELXS97* (Sheldrick, 2008 ▶); program(s) used to refine structure: *SHELXL97* (Sheldrick, 2008 ▶); molecular graphics: *CrystalStructure* (Rigaku/MSC, 2009 ▶); software used to prepare material for publication: *CrystalStructure*.

## Supplementary Material

Crystal structure: contains datablocks global, I. DOI: 10.1107/S1600536811011597/fj2404sup1.cif

Structure factors: contains datablocks I. DOI: 10.1107/S1600536811011597/fj2404Isup2.hkl

Additional supplementary materials:  crystallographic information; 3D view; checkCIF report

## Figures and Tables

**Table 1:** Hydrogen-bond geometry (Å, °) *Cg*3 is the centroid of the C13–C18 ring.

*D*—H⋯*A*	*D*—H	H⋯*A*	*D*⋯*A*	*D*—H⋯*A*
C2—H2⋯O1^i^	0.95	2.38	3.2906 (19)	161 (19)
C11—H11⋯N2	0.95	2.49	3.126 (2)	124
C11—H11⋯*Cg*3	0.95	2.98	3.7103 (17)	135
C17—H17⋯*Cg*3^ii^	0.95	2.98	3.6991 (17)	133

Symmetry codes: (i) 
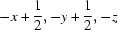
; (ii) 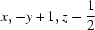.

**Fig. 1.**
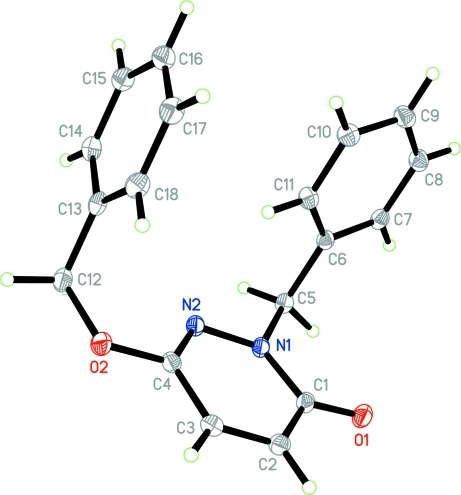
The asymmetric unit of the title compound, (I), with displacement ellipsoids drawn at the 30% probability level.

## supplementary crystallographic information

### Comment

Pyridazinone analogues have been reported to have a variety of biological activities, such as highly-selective anti-HIV agents (Loksha *et al.*, 2007), pesticide(Li *et al.*, 2005), highly herbicidal activity (Xu *et al.*, 2006). In order to discover further biologically active Pyridazinone analogues, the title compound, (I), was synthesized and its crystal structure determined (Fig. 1).

In a continuation of our studies on the crystal structures of Pyridazinone analogues (Liu *et al.*, 2005), we report here the synthesis and crystal structure of the title molecule, the central pyridazine ring forms dihedral angles of 77.08 (5)° and 84.62 (5)° with the two benzene rings, The dihedral angle between two benzene rings is 68.18 (4)°. The Crystal structure is stabilized by a weak intramolecular C—H···N hydrogen bond (Table 1), a weak intermolecular C—H···O hydrogen bond (Table 1), C—H···*Cg*π—ring (Table 1) and π-π stacking interactions where *Cg*(1)—*Cg*(1) (1/2 - *x*, 1/2 - *y*, 1 - *z*) is 3.6867 (10) Å [*Cg*(1) is the centroid of the N1,N2, C1—C4 ring] (Table 2).

### Experimental

Maleic hydrazide(0.56 g, 5 mmol), Benzyl chloride(1.52 g, 12 mmol) and K_2_CO_3_ (1.66 g, 12 mmol) were added to absolute ethanol(30 ml). The mixture was stirred in the room temperature for 6 h. The suspension was filtered and the residue was washed with absolute ethanol. The title compound was recrystallized from the mother solution and single crystals of (I) were obtained by slow evaporation.

### Refinement

All H atoms were placed in calculated positions, with C—H = 0.95 Å and C—H = 0.99 Å, and included in the final cycles of refinement using a riding model, with *U*_iso_(H) = 1.2Ueq(C).

### Crystal data

**Table:**

C_18_H_16_N_2_O_2_	*F*(000) = 1232
*M**_r_* = 292.33	*D*_x_ = 1.344 Mg m^−^^3^
Monoclinic, *C*2/*c*	Mo *K*α radiation, λ = 0.71073 Å
Hall symbol: -C 2yc	Cell parameters from 4881 reflections
*a* = 32.741 (4) Å	θ = 1.3–28.0°
*b* = 10.9198 (14) Å	µ = 0.09 mm^−^^1^
*c* = 8.1228 (10) Å	*T* = 113 K
β = 95.92 (2)°	Prism, colorless
*V* = 2888.6 (6) Å^3^	0.20 × 0.18 × 0.12 mm
*Z* = 8	

### Data collection

**Table:**

Rigaku Saturn CCD area-detector diffractometer	3448 independent reflections
Radiation source: rotating anode	2142 reflections with *I* > 2σ(*I*)
multilayer	*R*_int_ = 0.063
Detector resolution: 14.63 pixels mm^-1^	θ_max_ = 27.9°, θ_min_ = 1.3°
ω and φ scans	*h* = −43→41
Absorption correction: multi-scan *CrystalClear*	*k* = −14→14
*T*_min_ = 0.982, *T*_max_ = 0.989	*l* = −10→10
18031 measured reflections	

### Refinement

**Table:**

Refinement on *F*^2^	Primary atom site location: structure-invariant direct methods
Least-squares matrix: full	Secondary atom site location: difference Fourier map
*R*[*F*^2^ > 2σ(*F*^2^)] = 0.047	Hydrogen site location: inferred from neighbouring sites
*wR*(*F*^2^) = 0.116	H-atom parameters constrained
*S* = 0.95	*w* = 1/[σ^2^(*F*_o_^2^) + (0.0548*P*)^2^] where *P* = (*F*_o_^2^ + 2*F*_c_^2^)/3
3448 reflections	(Δ/σ)_max_ < 0.001
199 parameters	Δρ_max_ = 0.23 e Å^−^^3^
0 restraints	Δρ_min_ = −0.23 e Å^−^^3^

### Special details

**Table:**

Geometry. All e.s.d.'s (except the e.s.d. in the dihedral angle between two l.s. planes) are estimated using the full covariance matrix. The cell e.s.d.'s are taken into account individually in the estimation of e.s.d.'s in distances, angles and torsion angles; correlations between e.s.d.'s in cell parameters are only used when they are defined by crystal symmetry. An approximate (isotropic) treatment of cell e.s.d.'s is used for estimating e.s.d.'s involving l.s. planes.
Refinement. Refinement of *F*^2^ against ALL reflections. The weighted *R*-factor *wR* and goodness of fit *S* are based on *F*^2^, conventional *R*-factors *R* are based on *F*, with *F* set to zero for negative *F*^2^. The threshold expression of *F*^2^ > σ(*F*^2^) is used only for calculating *R*-factors(gt) *etc*. and is not relevant to the choice of reflections for refinement. *R*-factors based on *F*^2^ are statistically about twice as large as those based on *F*, and *R*- factors based on ALL data will be even larger.

### Fractional atomic coordinates and isotropic or equivalent isotropic displacement parameters (Å^2^)

**Table:**

	*x*	*y*	*z*	*U*_iso_*/*U*_eq_	
O1	0.20711 (3)	0.14159 (10)	0.06099 (12)	0.0315 (3)	
O2	0.20245 (3)	0.38939 (9)	0.62925 (12)	0.0282 (3)	
N1	0.18498 (4)	0.16470 (11)	0.31678 (14)	0.0221 (3)	
N2	0.18241 (4)	0.22441 (11)	0.46481 (14)	0.0229 (3)	
C1	0.20713 (5)	0.20250 (14)	0.19094 (18)	0.0241 (3)	
C2	0.22975 (5)	0.31536 (14)	0.22369 (18)	0.0257 (4)	
H2	0.2460	0.3470	0.1432	0.031*	
C3	0.22792 (5)	0.37578 (14)	0.36727 (18)	0.0253 (4)	
H3	0.2427	0.4499	0.3894	0.030*	
C4	0.20319 (5)	0.32523 (14)	0.48542 (17)	0.0231 (3)	
C5	0.15786 (4)	0.05874 (13)	0.29089 (17)	0.0232 (3)	
H5A	0.1559	0.0173	0.3982	0.028*	
H5B	0.1698	0.0000	0.2164	0.028*	
C6	0.11536 (5)	0.09390 (13)	0.21683 (17)	0.0222 (3)	
C7	0.09415 (5)	0.01638 (14)	0.10195 (17)	0.0257 (4)	
H7	0.1068	−0.0570	0.0701	0.031*	
C8	0.05468 (5)	0.04490 (15)	0.03337 (18)	0.0298 (4)	
H8	0.0406	−0.0082	−0.0461	0.036*	
C9	0.03585 (5)	0.15088 (15)	0.08102 (19)	0.0304 (4)	
H9	0.0088	0.1706	0.0347	0.036*	
C10	0.05657 (5)	0.22800 (14)	0.19643 (19)	0.0289 (4)	
H10	0.0436	0.3004	0.2299	0.035*	
C11	0.09614 (5)	0.20016 (14)	0.26341 (18)	0.0261 (4)	
H11	0.1102	0.2540	0.3417	0.031*	
C12	0.17631 (5)	0.34314 (15)	0.74918 (18)	0.0281 (4)	
H12A	0.1835	0.3852	0.8562	0.034*	
H12B	0.1818	0.2546	0.7665	0.034*	
C13	0.13136 (5)	0.36113 (13)	0.69709 (17)	0.0246 (4)	
C14	0.10296 (5)	0.28071 (14)	0.75401 (18)	0.0274 (4)	
H14	0.1122	0.2156	0.8260	0.033*	
C15	0.06137 (5)	0.29469 (14)	0.70671 (19)	0.0301 (4)	
H15	0.0422	0.2389	0.7454	0.036*	
C16	0.04764 (5)	0.39007 (14)	0.60290 (19)	0.0317 (4)	
H16	0.0191	0.3996	0.5698	0.038*	
C17	0.07560 (5)	0.47139 (15)	0.54761 (19)	0.0317 (4)	
H17	0.0663	0.5371	0.4768	0.038*	
C18	0.11706 (5)	0.45739 (14)	0.59503 (18)	0.0293 (4)	
H18	0.1360	0.5142	0.5575	0.035*	

### Atomic displacement parameters (Å^2^)

**Table:**

	*U*^11^	*U*^22^	*U*^33^	*U*^12^	*U*^13^	*U*^23^
O1	0.0356 (7)	0.0367 (7)	0.0229 (6)	−0.0022 (5)	0.0066 (5)	−0.0036 (5)
O2	0.0292 (6)	0.0304 (6)	0.0252 (6)	−0.0038 (5)	0.0037 (5)	−0.0070 (5)
N1	0.0246 (7)	0.0239 (7)	0.0177 (6)	−0.0016 (5)	0.0020 (5)	−0.0007 (5)
N2	0.0239 (7)	0.0255 (7)	0.0191 (6)	0.0017 (5)	0.0009 (5)	−0.0020 (5)
C1	0.0244 (8)	0.0282 (9)	0.0194 (7)	0.0034 (6)	0.0016 (6)	0.0022 (6)
C2	0.0243 (8)	0.0280 (9)	0.0247 (8)	−0.0005 (7)	0.0026 (6)	0.0038 (7)
C3	0.0223 (8)	0.0243 (8)	0.0288 (8)	−0.0007 (6)	0.0003 (7)	0.0015 (7)
C4	0.0221 (8)	0.0253 (8)	0.0213 (8)	0.0022 (6)	−0.0008 (6)	−0.0018 (6)
C5	0.0266 (8)	0.0211 (8)	0.0220 (7)	−0.0021 (6)	0.0029 (6)	−0.0001 (6)
C6	0.0248 (8)	0.0228 (8)	0.0193 (7)	−0.0019 (6)	0.0032 (6)	0.0028 (6)
C7	0.0300 (9)	0.0251 (8)	0.0225 (8)	−0.0044 (7)	0.0059 (7)	−0.0006 (6)
C8	0.0301 (9)	0.0333 (9)	0.0256 (8)	−0.0082 (7)	0.0006 (7)	−0.0015 (7)
C9	0.0274 (9)	0.0332 (9)	0.0298 (9)	−0.0029 (7)	−0.0008 (7)	0.0063 (7)
C10	0.0304 (9)	0.0259 (9)	0.0304 (9)	0.0019 (7)	0.0028 (7)	0.0021 (7)
C11	0.0283 (8)	0.0245 (8)	0.0247 (8)	−0.0005 (7)	−0.0011 (6)	−0.0018 (7)
C12	0.0322 (9)	0.0329 (9)	0.0194 (8)	−0.0013 (7)	0.0034 (7)	−0.0020 (7)
C13	0.0336 (9)	0.0216 (8)	0.0190 (7)	0.0002 (7)	0.0047 (6)	−0.0041 (6)
C14	0.0357 (9)	0.0240 (8)	0.0222 (8)	0.0001 (7)	0.0023 (7)	0.0001 (6)
C15	0.0327 (9)	0.0292 (9)	0.0289 (8)	−0.0041 (7)	0.0057 (7)	−0.0016 (7)
C16	0.0314 (9)	0.0340 (10)	0.0294 (9)	0.0024 (7)	0.0020 (7)	−0.0030 (7)
C17	0.0385 (10)	0.0275 (9)	0.0293 (9)	0.0056 (7)	0.0036 (7)	0.0030 (7)
C18	0.0345 (9)	0.0259 (9)	0.0283 (8)	−0.0012 (7)	0.0071 (7)	0.0034 (7)

### Geometric parameters (Å, °)

**Table:**

O1—C1	1.2476 (17)	C8—H8	0.9500
O2—C4	1.3647 (17)	C9—C10	1.385 (2)
O2—C12	1.4522 (18)	C9—H9	0.9500
N1—C1	1.3763 (19)	C10—C11	1.386 (2)
N1—N2	1.3780 (15)	C10—H10	0.9500
N1—C5	1.4605 (18)	C11—H11	0.9500
N2—C4	1.2959 (18)	C12—C13	1.502 (2)
C1—C2	1.448 (2)	C12—H12A	0.9900
C2—C3	1.347 (2)	C12—H12B	0.9900
C2—H2	0.9500	C13—C18	1.390 (2)
C3—C4	1.429 (2)	C13—C14	1.392 (2)
C3—H3	0.9500	C14—C15	1.385 (2)
C5—C6	1.507 (2)	C14—H14	0.9500
C5—H5A	0.9900	C15—C16	1.385 (2)
C5—H5B	0.9900	C15—H15	0.9500
C6—C11	1.391 (2)	C16—C17	1.383 (2)
C6—C7	1.392 (2)	C16—H16	0.9500
C7—C8	1.389 (2)	C17—C18	1.381 (2)
C7—H7	0.9500	C17—H17	0.9500
C8—C9	1.385 (2)	C18—H18	0.9500
			
C4—O2—C12	117.36 (12)	C10—C9—H9	120.1
C1—N1—N2	126.19 (12)	C8—C9—H9	120.1
C1—N1—C5	119.33 (12)	C9—C10—C11	120.34 (15)
N2—N1—C5	114.18 (11)	C9—C10—H10	119.8
C4—N2—N1	115.86 (12)	C11—C10—H10	119.8
O1—C1—N1	120.95 (14)	C10—C11—C6	120.47 (15)
O1—C1—C2	124.38 (14)	C10—C11—H11	119.8
N1—C1—C2	114.67 (13)	C6—C11—H11	119.8
C3—C2—C1	120.56 (14)	O2—C12—C13	113.20 (12)
C3—C2—H2	119.7	O2—C12—H12A	108.9
C1—C2—H2	119.7	C13—C12—H12A	108.9
C2—C3—C4	118.12 (14)	O2—C12—H12B	108.9
C2—C3—H3	120.9	C13—C12—H12B	108.9
C4—C3—H3	120.9	H12A—C12—H12B	107.8
N2—C4—O2	119.42 (13)	C18—C13—C14	118.68 (15)
N2—C4—C3	124.59 (13)	C18—C13—C12	121.80 (14)
O2—C4—C3	115.98 (13)	C14—C13—C12	119.51 (14)
N1—C5—C6	112.22 (12)	C15—C14—C13	120.64 (15)
N1—C5—H5A	109.2	C15—C14—H14	119.7
C6—C5—H5A	109.2	C13—C14—H14	119.7
N1—C5—H5B	109.2	C14—C15—C16	119.97 (16)
C6—C5—H5B	109.2	C14—C15—H15	120.0
H5A—C5—H5B	107.9	C16—C15—H15	120.0
C11—C6—C7	118.77 (14)	C17—C16—C15	119.76 (16)
C11—C6—C5	121.93 (14)	C17—C16—H16	120.1
C7—C6—C5	119.28 (14)	C15—C16—H16	120.1
C8—C7—C6	120.80 (15)	C18—C17—C16	120.19 (15)
C8—C7—H7	119.6	C18—C17—H17	119.9
C6—C7—H7	119.6	C16—C17—H17	119.9
C9—C8—C7	119.86 (15)	C17—C18—C13	120.74 (15)
C9—C8—H8	120.1	C17—C18—H18	119.6
C7—C8—H8	120.1	C13—C18—H18	119.6
C10—C9—C8	119.75 (15)		
			
C1—N1—N2—C4	−0.3 (2)	C11—C6—C7—C8	0.7 (2)
C5—N1—N2—C4	−173.91 (12)	C5—C6—C7—C8	178.90 (13)
N2—N1—C1—O1	−179.53 (13)	C6—C7—C8—C9	−0.9 (2)
C5—N1—C1—O1	−6.3 (2)	C7—C8—C9—C10	0.2 (2)
N2—N1—C1—C2	0.8 (2)	C8—C9—C10—C11	0.5 (2)
C5—N1—C1—C2	174.04 (12)	C9—C10—C11—C6	−0.6 (2)
O1—C1—C2—C3	179.70 (14)	C7—C6—C11—C10	0.0 (2)
N1—C1—C2—C3	−0.6 (2)	C5—C6—C11—C10	−178.11 (14)
C1—C2—C3—C4	0.1 (2)	C4—O2—C12—C13	−72.05 (17)
N1—N2—C4—O2	−179.59 (11)	O2—C12—C13—C18	−28.7 (2)
N1—N2—C4—C3	−0.3 (2)	O2—C12—C13—C14	152.30 (13)
C12—O2—C4—N2	−3.1 (2)	C18—C13—C14—C15	1.5 (2)
C12—O2—C4—C3	177.58 (12)	C12—C13—C14—C15	−179.46 (14)
C2—C3—C4—N2	0.4 (2)	C13—C14—C15—C16	−0.6 (2)
C2—C3—C4—O2	179.72 (13)	C14—C15—C16—C17	−0.3 (2)
C1—N1—C5—C6	−88.39 (16)	C15—C16—C17—C18	0.3 (2)
N2—N1—C5—C6	85.65 (14)	C16—C17—C18—C13	0.7 (2)
N1—C5—C6—C11	−38.55 (18)	C14—C13—C18—C17	−1.6 (2)
N1—C5—C6—C7	143.33 (13)	C12—C13—C18—C17	179.41 (14)

### Hydrogen-bond geometry (Å, °)

**Table:**

Cg3 is the centroid of the C13–C18 ring.

**Table:**

*D*—H···*A*	*D*—H	H···*A*	*D*···*A*	*D*—H···*A*
C2—H2···O1^i^	0.95	2.38	3.2906 (19)	161 (19)
C11—H11···N2	0.95	2.49	3.126 (2)	124
C11—H11···Cg3	0.95	2.98	3.7103 (17)	135
C17—H17···Cg3^ii^	0.95	2.98	3.6991 (17)	133

Symmetry codes: (i) −*x*+1/2, −*y*+1/2, −*z*; (ii) *x*, −*y*+1, *z*−1/2.

### Table 2 Comparative geometrical parameters (Å) for selected Cg—Cg Π stacking interaction, Cg1 is the centroid of the N1,N2,C1-C4 ring (Symmetry codes: 1/2-X,1/2-Y,1-Z).

**Table:**

CgI—CgJ	Cg—Cg(Å)	CgIPerp(Å)	CgjPerp(Å)	Slippage(Å)
Cg1—Cg1	3.6867 (10)	3.224	3.224	1.789
